# A Mixed-Methods Evaluation of a Novel, Augmented Reality Educational App for Asthma

**DOI:** 10.1177/29941520251372952

**Published:** 2025-09-03

**Authors:** David V. Swetland, Heidi J. Sucharew, Bradley Cruse, Michael Glisson, Francis J. Real

**Affiliations:** ^1^ Department of Pediatrics, Division of Pulmonary Medicine, Tulane University School of Medicine, New Orleans, Louisiana, USA.; ^2^ Department of Emergency Medicine, University of Cincinnati College of Medicine, Cincinnati, Ohio, USA.; ^3^ Digital Experience Technologies, Cincinnati Children’s Hospital Medical Center, Cincinnati, Ohio, USA.; ^4^ Department of Pediatrics, University of Cincinnati College of Medicine, Cincinnati, Ohio, USA.; ^5^ Division of General and Community Pediatrics, Cincinnati Children’s Hospital Medical Center, Cincinnati, Ohio, USA.

**Keywords:** gamification, mhealth, patient education, augmented reality, asthma education

## Abstract

**Background:**

Asthma education is an important component of asthma management and control. Technology-based approaches via mobile health applications have been studied in many different diseases, including asthma. Innovative educational approaches, such as augmented reality (AR) serious games, may be effective and appealing to caregivers of children with asthma, with the goal of improving asthma control.

**Objectives:**

The purpose of this mixed-methods study was to assess the usage, acceptability, and feasibility of an AR component to the *CHANGE-Asthma* (Clinic, Home, and On-the-Go Education for Asthma) app, an educational bundle using serious games to support self-directed learning.

**Methods:**

This was an explanatory, sequential mixed-methods study. Quantitative data consisted of usage time within the app. Qualitative data consisted of semi-structured interviews evaluated via thematic analysis to identify recurring themes related to their experiences.

**Results:**

Time spent within the app decreased after the first week. The mean total time spent within the app was 8.6 min over an average of 14 weeks (range 9–19 weeks). Themes identified from thematic analysis included perceptions of the app as helpful to both participants and others with AR supporting asthma management skills. Some users reported technological challenges with using AR.

**Conclusions:**

Despite low usage of the *CHANGE-Asthma* app, caregivers held an overall positive view of the app and its educational features. AR was well received as a tool for deliberate practice but limited by difficulty with utilizing the AR feature on their personal devices.

## Introduction 

Asthma is a common chronic disease that results in substantial patient morbidity and significant lifetime costs to the health care system.^1^ Asthma education during routine clinical care generally consists of in-person demonstrations, printouts, and pamphlets with variable success in teaching fundamental management skills.^2,3^ One successful approach to asthma education has been the use of individualized education, such as asthma action plans (AAP), which have demonstrated improved health outcomes.^4^ Technology-based strategies, such as mobile health (mHealth) applications (apps), have been a topic of research emphasizing self-management strategies and education; these approaches have demonstrated mixed results on impacting quality of life and asthma control measures when compared to standard care.^5–7^ MHealth development still remains in its infancy, with little high-level evidence to support its use to date.^8^ More recently, apps using elements of gamification have shown promising results for pediatric asthma with improved control, knowledge, and quality of life.^6,9^ Serious games are games with a pedagogical purpose that target knowledge and skill. With continued advances in technology, assessing novel approaches to education is critical to determine what approaches might be most acceptable to patients and improve outcomes.^10,11^

Augmented reality (AR), a combination of real-world environments and computer-generated information visualized via a digital device, provides a novel opportunity for asthma education. Apple and Google have integrated AR features into their smartphone operating systems since 2017 and 2018, respectively. AR holds great potential for patient education, as it can use gamification to support deliberate practice and structured activities with explicit goals of developing expertise and enhancing understanding.^12^ Clinical education utilizing AR has been previously assessed for pediatric diabetes education, demonstrating improved knowledge and satisfaction with management of this disease.^13^ To our knowledge, AR has not been previously applied to outpatient, pediatric asthma education.

Innovative educational approaches utilizing technology may appeal to modern patients and caregivers and, ultimately, improve asthma control. Novel technologies, such as AR, may support some degree of education that may be missing compared to the in-person clinical model. It is important that such approaches consider the application of adult-learning theory to best support engagement and learning. Thus, we proposed to evaluate a novel educational strategy that leverages AR technology in consideration of adult-learning theory for caregiver-focused pediatric asthma education.

A pilot study of *CHANGE-Asthma* (Clinic, Home, and On-the-Go Education for Asthma) focused on education for asthma management was completed at the Cincinnati Children’s Hospital Medical Center (CCHMC) Pediatric Primary Care Center from September 2017 to November 2017.^14^ The study evaluated changes in childhood Asthma Control Test (cACT) scores over the course of a 3-month period using an earlier version of the app. Results showed an improvement in cACT scores over time within the intervention group that was exposed to the *CHANGE-Asthma* app with a positive dose-dependent response (that is, those who used the app more had a higher change in cACT scoring) despite low average usage time. Caregiver assessment of the app was not formally studied qualitatively at that time. The purpose of this mixed-methods study was to assess the feasibility and real-world usage of an updated version of *CHANGE-Asthma* that included novel AR features and to assess caregivers’ perspectives on this innovative approach to education.

## Methods

### Setting and participants

The study population consisted of caregivers (18 years and older) of children ages 4–11 years who had intermittent, mild persistent, or moderate persistent asthma. All caregivers had a child who was seen in telehealth clinics held by a member of the Division of Pulmonary Medicine at CCHMC between March and August 2020. Inclusion criteria included (1) patients using a daily, metered-dose inhaler (MDI) controller medication, (2) being an English-speaking caregiver, and (3) having possession of a smartphone device using either iOS or Android operating systems. Exclusion criteria included (1) patients using a dry powder inhaler controller medication, as education was not present in the application at that time, and (2) caregivers not being legally able to provide informed consent. Participants were recruited through either telephone or e-mail for participation. This study was approved by the CCHMC Institutional Review Board (IRB#: 2020-0097).

### Application development

**FIG. 1.**
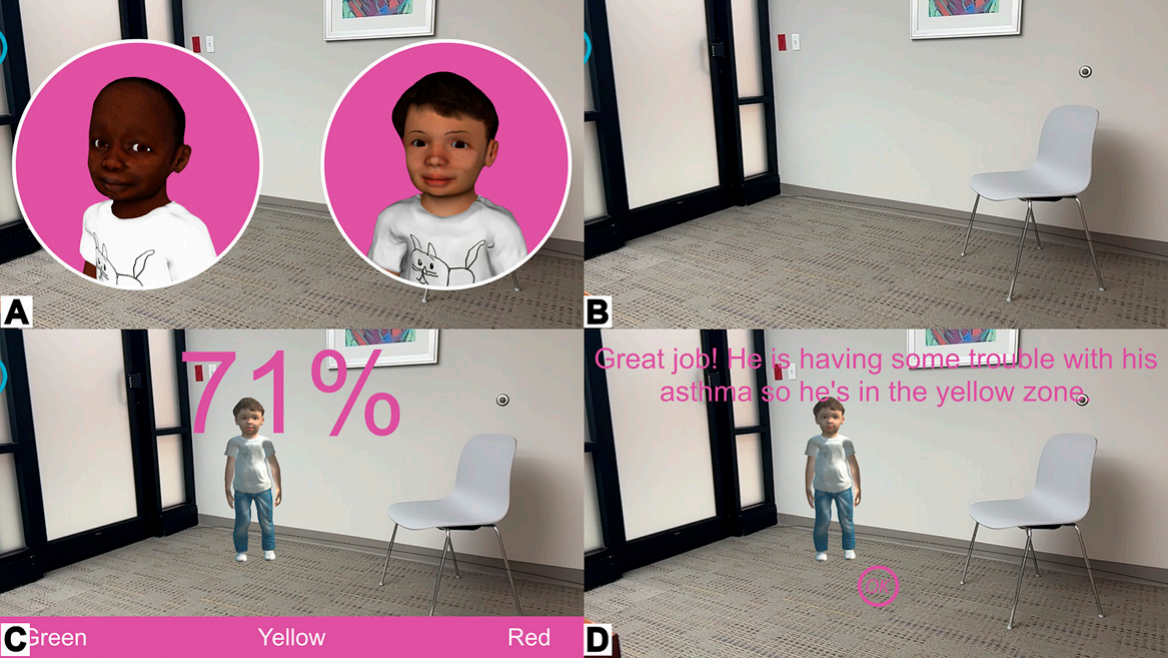
**(A)** When loading into the AR feature, users are offered the ability to select one of two avatar characters. **(B)** The AR software built into iOS and Android detects a flat surface and encircles it with a black circle for available locations to place the avatar. The avatar remained anchored to this location, no matter where the camera was focused. **(C)** Users receive a case and observe the avatar demonstrating specific pulmonary symptoms via audio and visual cues. Users select what zone of the asthma action plan the avatar is demonstrating. Cases are randomized to provide variation. There is a cumulative score presented on the screen as a percentage. **(D)** Users receive immediate feedback on their selection. AR, augmented reality.

This study evaluated an iterative update to a previously developed and evaluated educational mHealth app entitled *CHANGE-Asthma,* which was developed at CCHMC. The app consisted of three components: (1) educational videos, including proper MDI usage; (2) a personalized AAP; and (3) serious games reinforcing key concepts of asthma management. These serious games challenged families to select the correct medication to give to an animated patient based on the visual presentation of the avatar (e.g., work of breathing).^15^ To enhance the personalization and immersive nature of serious games, AR was incorporated into the *CHANGE-Asthma* app utilizing internal AR software in iOS and Android devices. These AR features expanded the serious game functionality to allow users to place the animated patient in any environment, including their own home. The app used the infrared camera to recognize and encircle a flat surface for placement of the avatar, which needed to be done every time the AR portion of the app was loaded to ensure the avatar would appear near the user. The process of placing the avatar in a room via AR is further described in Figure 1. This educational approach aligned with the conceptual framework of self-directed learning that relies on a caregiver’s self-management, self-monitoring, and motivation to learn.^16^ The serious games also incorporated principles of deliberate practice by providing immediate feedback to users.^12,17^

### Design and procedures

This study used a mixed-methods approach with an explanatory, sequential design that was meant to assess caregiver perspectives on the incorporation of AR into *CHANGE-Asthma,* focusing on feasibility and usability.^18^ First, we collected quantitative data related to app usage. Next, we conducted semi-structured interviews with caregivers based on the findings related to usage to obtain perspectives on the AR serious games. The combined data from the two converging designs presented a unified evaluation of this novel approach to asthma education.

Demographic data were obtained at enrollment. Study data were collected and managed using the REDCap (Research Electronic Data Capture) electronic data capture tools hosted at CCHMC.^19,20^ REDCap is a secure, web-based application designed to support data capture for research studies.

Primary caregivers downloaded the app to their device at enrollment. The data monitored included time, in seconds, while using the app; this feature was explicitly discussed with the caregiver during the consent procedure. Data were downloaded in weekly batches for all patients. The app created a unique ID number that protected the identity of participants in the study. This ID number was randomly generated upon first opening and was paired with the REDCap study ID.

Participants were asked to undergo a semi-structured, individual qualitative interview led by a single author (D.V.S.). Audio of the interviews was recorded and subsequently transcribed into Microsoft Word®. The researchers used purposeful sampling from participants to cover the range of usage within the app. Interviews were performed and assessed via thematic analysis until theoretical sufficiency was reached, based on repetition of themes within the data.^21^

### Instrument design

Qualitative interview questions were developed by the study team prior to the initiation of the study. The chosen questions were reviewed by educational experts at CCHMC as well as members of the Divisions of General & Community Pediatrics and Pulmonary Medicine. Initial survey responses and early app usage data were evaluated and used to develop sub-questions and new questions, as often occurs with qualitative interviews. A copy of the interview guide can be found in Supplementary Table S1.

### Analysis

Descriptive statistics were calculated using SPSS® 26 statistical software. The interview data were analyzed using ATLAS.ti® data analysis software. Thematic analysis was used to identify themes within the data.^21^ Thematic analysis was completed by two authors (D.V.S., F.J.R.) and focused on the effectiveness of the technology and educational material.

## Results

### Quantitative findings

A total of 28 (32%) out of 88 eligible caregivers agreed to be a part of the study and completed the enrollment process. Caregiver and patient characteristics are shown in Table 1.

**Table 1.** Baseline Demographic Data

	All participants^a^	Interview participants
Parent age (years, mean ± SD)	37.8 ± 6.8	37.4 ± 4.5
Parent sex, *n* (%)		
Female	26 (96)	14 (93)
Male	1 (4)	1 (7)
Parent race, *n* (%)		
Black or African American	5 (19)	1 (7)
White or Caucasian	21 (78)	13 (87)
Mixed Race	1 (4)	1 (7)
Child age (years, mean ± SD)	6.9 ± 2.1	7.1 ± 2.3
Child sex, *n* (%)		
Female	9 (33)	7 (47)
Male	18 (67)	8 (53)
Child race, *n* (%)		
Black or African American	5 (19)	1 (87)
White or Caucasian	20 (74)	13 (7)
Mixed Race	1 (4)	1 (7)
Other	1 (4)	0 (0)
Smartphone operating system, *n* (%)		
Apple iOS	17 (63)	9 (60)
Google Android	10 (37)	6 (40)

^a^ One participant did not complete demographic survey data.

The average length of data collection per caregiver was 14 weeks (*n* = 20, range 9–19 weeks). The average total time of usage during the first week was 5.5 min (range 1.7–17.5 min). The average total time of usage over the course of the study was 8.6 min (range 3.8–24.7 min) per caregiver. Ten (50%) caregivers logged time in the app for two or more weeks after the first week. Three (15%) caregivers of the caregivers did not use the app after the first week.

### Qualitative findings

Fifteen qualitative interviews were held, and quotes for theme development are included in Table 2. During the interviews, several common themes emerged based on caregiver usage of the app. In general, participants held an overall positive view of the app. The most common terms used were “*helpful*,” “*informative*,” and “*easy-to-use*.” The app was used until saturation, that is, until the caregiver felt comfortable with their level of knowledge, whether from previous experience or from the app. The app was suggested as a strong educational tool for those new to asthma or family members who have not been present for in-person asthma education. The use of AR technology found within the app was a positive feature for reinforcing and reiterating the important parts of acute management. Nevertheless, there were issues with both app functionality and use of new technology, using terms such as “glitchy” and “user error.”

**Table 2.** Qualitative Feedback from Interviews (*n* = 15)

Theme	Example quotation
General view as helpful	“But I think that it’s really helpful, because then you can, you can get this out at your leisure and you know, at the time of day that you can focus better or whatever.” (Participant [P] 8) “We just clicked on it. It was pretty straightforward. The answers were obvious based on how the avatar’s breathing was doing. It seemed like a good learning experience.” (P3) “But I’ve learned something that I didn’t know before. So, it can’t harm [someone] to go over everything that you think you know. It’s just going over the procedures and what needs to be done in case something goes wrong.” (P7) “Having this at our fingertips at the beginning, when we didn’t know what to do… would have been helpful.” (P14)
Usage based on educational saturation	“After I used it frequently in the beginning, it probably didn’t help me anymore. Because it taught me… what I needed to know.” (P10) “I also felt like I did a lot all at once… and then I was like, ‘okay, did I do everything? Have I experienced everything?’ and I felt like I had.” (P11) “We have a good action plan in place and I’m comfortable with all this medication and comfortable in how to treat him.” (P15) “I think it’s just, you know, where we’ve been dealing with it for so many years. So, we’re kind of already educated in it.” (P12)
Prospective educational targets	“I think it would be super handy for first time parents going through asthma, and a nice tool to kind of teach their child about it as well.” (P1) “I really feel like maybe my husband could have benefitted from watching the videos, because he’s not at all of the appointments.” (P6) “I didn’t even think to do this for my mother-in-law… but she watches my kids and I think that it would be really helpful for her to see it.” (P8)
Augmented reality allowed for practice	“You feel like it’s real, it’s a real situation. It’s not just written on paper… it’s almost like you are in the hospital having a training about something that’s happening to asthma patients.” (P7) “It was like a kind of practice… making sure that we knew what to do in a situation… I especially will have some anxiety surrounding that thinking, like, second guessing myself. So, it’s nice to just have that reinforcement.” (P5) “Because I started with a certain base of knowledge, just from my own experience, but I don’t have a lot of knowledge of watching my son, of being an observer. And it let me be an observer.” (P11) “There was repetition… that helped reinforce the process.” (P11)
Functionality challenges with augmented reality	“I’m not a fan of like the holding the phone out to like, line up a child and in the view, because it’s, it’s kind of a little wonky each time I go back and do that. And it has you like, wherever you set the character, you have to make sure that you go back to that specific spot each time.” (P1) “Sometimes we couldn’t get the character to come up to utilize it properly, or it could just be our house.” (P5) “I couldn’t get (the avatar) to work. And I don’t consider myself non-tech savvy.” (P14)

## Discussion

Through this mixed-methods study, we evaluated an asthma-focused, educational app with usage analytics and aimed to understand caregivers’ insights with qualitative interviewing. We were able to assess caregiver attitudes toward the low usage rates and utilizing AR features within smartphone devices.

When distributing technological interventions such as ours, it is imperative to assess usability and analytics early so challenges related to deployment can be evaluated and iterative improvements can be made. Overall usage was low, which was similar to previous evaluations of the *CHANGE-Asthma* app.^14^ Our usage rates dropped notably after the first week of enrollment. Many caregivers would reengage with the app either the week of the interview or survey e-mail, suggesting reminders may offer a benefit. Reminders, such as notifications, for educational apps will need to be further evaluated to determine the appropriate time and place to improve usage, as has been done with non-mHealth apps.^22^ Participants generally brought up their low usage in the open-ended interview format without prompting. Caregivers saw it as a lack of motivation based on self-monitoring of their knowledge level, a key component of self-directed learning. Several brought up that they “forgot” about the app between interactions with the research team. Even with the usage in this sample group being low, there was general support for the updated *CHANGE-Asthma* app; a few brought up situations where they may use it again. Most caregivers suggested a population, such as an extended family member, who could benefit from the framework of self-directed learning from an app-based educational bundle. This was a sentiment even in those who did not find the app helpful for their own learning.

Many caregivers were interested in utilizing the novel AR technology. The AR feature allowed caregivers to partake in medical simulation-based education, something that has been utilized for and evaluated in health care professionals.^23^ Caregivers discussed their use of AR in ways that were reminiscent of Ericsson et al.’s discussion of deliberate practice in developing expertise and enhancing understanding.^12^ The repetitive nature of the AR games offered repeated task performance for reinforcement of skills and knowledge, and caregivers found it an important aspect of their practice. These statements were made despite the low time within the app and some reported concerns and difficulties with the novel AR feature. Caregivers specifically brought up the design choice of the spatially anchored avatar as something they did not like. The spatially anchored avatar stayed anchored to one specific point on a horizontal surface in the physical room of the user, displayed in AR, as opposed to constantly in frame like a standard video. From a technical standpoint, some caregivers found the action of placing the avatar in a scene to be difficult. Two issues arose. Sometimes there was no possible room to place the avatar, and sometimes the placement location was not on the ground. Users would have to exit the AR section to reset the available placement location. Moreover, several participants were unable to utilize the AR features because of older hardware not receiving operating system updates that included the built-in AR features of iOS or Android.

This study will be beneficial in future, iterative updates to the *CHANGE-Asthma* app by utilizing the principles of user-centered design.^24^ This version of the app observed and analyzed the use of the AR features and tested them directly with the end-users. Future iterative updates can focus on resolving errors of avatar placement, evaluating design alternatives that caregivers prefer, and testing with a wider user base.

There are limitations to this study. First, the sample size was small for quantitative data, which limits generalizability and may not be representative of the population of those engaged in subspecialty care for persistent asthma. Nevertheless, the size was appropriate for a qualitative evaluation. Second, self-selection bias may have contributed to enrollment, as those with increased interest in AR or technology-based education may have chosen to enroll. However, when conducting usability testing of a new technology, an increased interest in technology may have allowed for more meaningful insights by users.

## Conclusion

App-based, educational mHealth programs continue to be upheld as a potential next step in personalized medical management. With this study, we were able to obtain caregiver insights of an educational mHealth app utilizing AR while observing real-world usage statistics. The *CHANGE-Asthma* app was well received despite minimal prolonged usage. Participants felt comfortable in their knowledge of asthma management but felt that others could benefit from the app. Caregivers noted functionality challenges with AR limiting universal appeal. These findings will help improve the *CHANGE-Asthma* app in future iterative updates. Larger studies will need to be utilized to evaluate clinical outcomes, patient and caregiver knowledge, and how to improve usage for mHealth apps.

## Ethical Approval

The study was approved by the Cincinnati Children’s Hospital Medical Center Institutional Review Board.

## Abbreviations Used

AAP: Asthma Action Plans
Apps: Applications
AR: Augmented Reality
cACT: Childhood Asthma Control Test
CCHMC: Cincinnati Children’s Hospital Medical Center
CHANGE-Asthma: Clinic, Home, and On-the-Go Education for Asthma
MDI: Metered-Dose Inhaler
mHealth: Mobile Health
REDCap: Research Electronic Data Capture
