# Supplementary material for: A Mixed-Methods Evaluation of a Novel, Augmented Reality Educational App for Asthma
Source: J Med Ext Real. 2025 Sep 3;2(1):183–9. doi: 10.1177/29941520251372952 (PMC13387246; doi:10.1177/29941520251372952)
Supplement: Supplementary file 1 [file 29941520251372952_supp_tables1.docx]

**Table E1: Interview Questionnaire**

**Overall Impression**

1. What are your overall impressions of the *CHANGE-Asthma* app?

2. What did you like the most? What did you like the least?

3. What 2 words might you use to describe the *CHANGE-Asthma* app?

**Augmented Reality Evaluation**

1. Tell me about the experience of using the Augmented Reality games.

2. Did the Augmented Reality games teach you about your child’s asthma? If so, how?

a. If it was helpful why?

b. If it was not helpful, why not?

**App Usage**

1. How often did you use the app?

2. Do you anticipate using the *CHANGE-Asthma* app in the future?

a. If yes, are there certain times it might be most useful?

b. If no, why not?

3. If you could make one significant change to *CHANGE-Asthma*, what change would you make?

**Recommendation/Telehealth**

1. Would you recommend that other parents download the app to learn about their child’s asthma?

a. If yes, why?

b. If no, why not?

2. What did you think of the pairing of the app download with your telehealth visit?

a. Do you see the app playing a role in in-person visits as well?
